# Predicting and Monitoring Immune Checkpoint Inhibitor Therapy Using Artificial Intelligence in Pancreatic Cancer

**DOI:** 10.3390/ijms252212038

**Published:** 2024-11-09

**Authors:** Guangbo Yu, Zigeng Zhang, Aydin Eresen, Qiaoming Hou, Farideh Amirrad, Sha Webster, Surya Nauli, Vahid Yaghmai, Zhuoli Zhang

**Affiliations:** 1 Department of Biomedical Engineering, University of California, Irvine, CA 92617, USA; guangboy@uci.edu; 2 Department of Radiological Sciences, University of California, Irvine, CA 92868, USA; zigengz@hs.uci.edu (Z.Z.); aeresen@hs.uci.edu (A.E.); qiaominh@hs.uci.edu (Q.H.); vyaghmai@hs.uci.edu (V.Y.); 3 Chao Family Comprehensive Cancer Center, University of California Irvine, Irvine, CA 92612, USA; 4 Department of Biomedical and Pharmaceutical Sciences, Harry and Diane Rinker Health Science Campus, Chapman University, Irvine, CA 92618, USA; amirrad@chapman.edu (F.A.); shwebster@chapman.edu (S.W.); nauli@chapman.edu (S.N.); 5 Department of Medicine, University of California Irvine, Irvine, CA 92868, USA; 6 Department of Pathology and Laboratory Medicine, University of California Irvine, Irvine, CA 92617, USA

**Keywords:** PDAC, immunotherapy, immune checkpoint inhibitors, radiomics, artificial intelligence, deep learning, machine learning

## Abstract

Pancreatic cancer remains one of the most lethal cancers, primarily due to its late diagnosis and limited treatment options. This review examines the challenges and potential of using immunotherapy to treat pancreatic cancer, highlighting the role of artificial intelligence (AI) as a promising tool to enhance early detection and monitor the effectiveness of these therapies. By synthesizing recent advancements and identifying gaps in the current research, this review aims to provide a comprehensive overview of how AI and immunotherapy can be integrated to develop more personalized and effective treatment strategies. The insights from this review may guide future research efforts and contribute to improving patient outcomes in pancreatic cancer management.

## 1. Introduction

Pancreatic ductal adenocarcinoma (PDAC) is a significant global health challenge, ranking as the fourth leading cause of cancer-related mortality, causing 466,003 deaths annually worldwide. In the United States, the 5-year relative survival rate for pancreatic cancer is alarmingly low, standing at merely 12% [1]. Forecasts from 2021 suggest that by 2040, the number of deaths caused by pancreatic cancer will surpass those caused by colorectal cancer, making it the second leading cause of cancer-related death in the United States [2]. Since it is predominantly diagnosed at advanced stages, PDAC treatment options are limited, with surgical intervention being the traditional approach. However, the efficacy of surgery for late-stage PDAC is significantly compromised due to delayed diagnosis, which hinders resectability and is compounded by a high incidence of inoperable disease. The early detection of PDAC is challenging due to its asymptomatic nature in the initial stages and the lack of specific biomarkers, which contributes to the high mortality rate associated with this cancer [3].

In cancer immunotherapy (CIT), patients often exhibit atypical tumor response patterns, most notably “pseudoprogression” [4]. This phenomenon, characterized by the transient enlargement of tumors or the emergence of new lesions due to T-cell infiltration, diverges from traditional criteria like RECIST, which would typically interpret such changes as disease progression. Consequently, the early and accurate assessment of CIT responses becomes challenging, as existing modified criteria like RECIST, iRECIST, and imRECIST [5,6,7] do not fully account for tumor heterogeneity, the nuances of pseudoprogression, and immune-related mixed response patterns. This complexity complicates the prediction of clinical outcomes, such as progression-free survival and overall survival. Additionally, the immunosuppressive microenvironment of PDAC further diminishes the efficacy of immune checkpoint inhibitors (ICIs), underscoring the urgency of developing effective treatment strategies.

In contrast, artificial intelligence (AI) presents a promising alternative by leveraging conventional medical imaging data to identify novel biomarkers for monitoring pathophysiological responses to CIT. AI-based algorithms, known for their superior efficiency compared to manual methods in cancer imaging, facilitate the advanced quantification of tumor burden and treatment response [8,9]. By integrating radiomics analysis with sophisticated statistical methods, these AI frameworks aim to extract descriptive imaging biomarkers that correlate with histological tumor markers, thereby enhancing the prediction of treatment outcomes. The expected correlation between AI-derived imaging biomarkers and histological findings holds the potential to refine prognosis and accurately predict responses to treatments such as anti-PD1, anti-CTLA-4, or combination therapy, thereby surpassing the limitations of current assessment methodologies.

In this review, we examine the role of ICIs in the treatment of PDAC, focusing on the challenges posed by the tumor’s immunosuppressive microenvironment and strategies to enhance the efficacy of these therapies. We also explore the emerging role of artificial intelligence (AI), particularly in the early detection of PDAC and the monitoring of immunotherapy outcomes. By integrating insights from both immunotherapy and AI, this review provides a comprehensive overview of current advancements and future directions in PDAC management, highlighting the potential of AI-driven approaches to improve diagnostic accuracy, personalize treatment, and ultimately enhance patient outcomes.

## 2. ICI Mechanism and Treatment for PDAC

**Figure 1:**
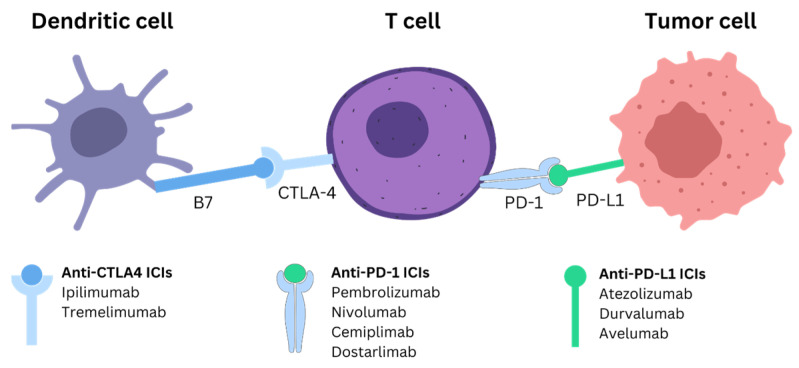
Mechanism of action of immune checkpoint inhibitors in cancer treatment. It shows the interaction between dendritic cells, T-cells, and tumor cells, focusing on immune checkpoint pathways. Anti-CTLA4 ICIs (e.g., Ipilimumab, Tremelimumab) block the CTLA-4/B7 interaction, activating T-cells. Anti-PD-1 (e.g., Pembrolizumab, Nivolumab) and anti-PD-L1 (e.g., Atezolizumab, Durvalumab) ICIs disrupt the PD-1/PD-L1 interaction, restoring T-cell function to attack tumor cells.

ICIs function by targeting and inhibiting specific immune checkpoint proteins that regulate the immune system, preventing overactivation and autoimmunity (Figure 1). The primary mechanism involves blocking the interaction between checkpoint proteins and their ligands, which suppresses the immune response against tumors [10,11]. For instance, PD-1, an immune checkpoint protein, modulates T-cell activity in peripheral tissues through its interaction with PD-L1 and PD-L2. Blocking PD-1 or its ligands with ICIs prevents the “off” signal that would normally reduce T-cell activity, thus sustaining an active immune response against tumor cells [12,13,14,15]. Similarly, CTLA-4, another checkpoint protein, attenuates the activation of naïve and memory T-cells by binding to the ligands B7-1 (CD80) and B7-2 (CD86) on antigen-presenting cells. By inhibiting CTLA-4, ICIs enhance T-cell activation and proliferation, thereby boosting the immune response against cancer cells [12,16]. Figure 1 illustrates the mechanism of ICIs and the commonly used ICIs for each type.

ICIs have shown significant promise in treating various solid tumors [17], particularly those classified as immune “hot” tumors, which are characterized by high immune cell infiltration and a high tumor mutational burden (TMB) [18]. However, PDAC presents unique challenges for ICIs due to its typically “cold” tumor microenvironment, which lacks sufficient immune cell infiltration and exhibits low immunogenicity, making it less responsive to immunotherapy [19,20,21]. Despite these challenges, certain subgroups of tumors, such as those with deficient mismatch repair (dMMR) [22] or high microsatellite instability (MSI-H) [23,24], have shown better responses to ICIs. In a multi-institutional analysis, patients with MSI/dMMR PDAC treated with ICIs showed a median progression-free survival (PFS) of 26.7 months and a high disease control rate, suggesting that ICIs can be effective in this subgroup [25]. Additionally, patients with a high tumor mutational burden (TMB) also benefit from ICI therapy, with a significantly improved overall survival (OS) and time to treatment discontinuation (TTD) compared to those with low TMB [26]. Transforming immune “cold” tumors, typically less responsive to ICIs due to their low immunogenicity and immune escape mechanisms, into “hot” tumors is a potential strategy to enhance immunotherapy efficacy [27,28]. Combination therapy is another promising approach. For example, combining radiotherapy with ICIs can induce the abscopal effect, enhancing systemic antitumor responses and potentially improving the efficacy of treatments for PDAC [29].

Overall, while ICIs hold promise, their application in PDAC requires further research to overcome the unique challenges posed by the tumor’s microenvironment and to identify effective combination strategies that can re-engage immune responses for better clinical outcomes.

### 2.1. PD-1/PD-L1

PD-1 is an immune checkpoint expressed on activated T-cells. Upon binding to its ligand PD-L1, it inhibits T-cell activity and promotes immune tolerance, making it a target for immunotherapy in various cancers [30]. However, in PDAC, PD-1/PD-L1 blockade monotherapy has shown limited efficacy due to the tumor’s immunosuppressive microenvironment and intrinsic non-immunogenic nature [31]. More effective treatments are needed to address these challenges.

A study explored the use of pembrolizumab, an anti-PD-1 ICI, in the treatment of pancreatic cancer with mismatch repair deficiency. The findings demonstrated that PD-1 blockade could be effective in PDAC patients with high mutational burdens caused by mismatch repair defects, resulting in a substantial immune response and improved clinical outcomes [22]. However, more universal treatment approaches are needed. A study by Moral et al. reveals that Group 2 innate lymphoid cells (ILC2s) enhance the efficacy of PD-1 blockade in PDAC by activating tissue-specific tumor immunity, presenting a novel approach to improve immunotherapy outcomes [32]. Additionally, a recent study demonstrated that modulating the intratumor microbiome with a probiotic engineered to disrupt bacterial iron respiration enhances the efficacy of PD-L1 blockade in pancreatic cancer by reducing immunosuppressive signals and improving cytotoxic T lymphocyte infiltration [33].

Ongoing clinical trials are exploring various strategies to overcome the immunosuppressive environment of PDAC (Table 1). One such trial compared the efficacy of niraparib combined with either nivolumab (anti-PD-1) or ipilimumab (anti-CTLA-4) in patients with advanced pancreatic cancer. The study found that niraparib plus ipilimumab resulted in a superior progression-free survival rate at 6 months (PFS6) of 59.6% compared to 20.6% for niraparib plus nivolumab [34]. Another phase 1 clinical trial has been initiated to test the safety, preliminary efficacy, and biomarkers of response to the combination of Trametinib (MEK inhibitor), Ruxolitinib (JAK2/STAT3 inhibitor), and Retifanlimab (PD-1 inhibitor) in patients with metastatic PDAC who have progressed on prior therapy. This combination aims to overcome ICI resistance by enhancing CD8^+^ T-cell cytotoxicity and antitumor responses [35]. In another phase I trial, personalized RNA neoantigen vaccines, combined with anti-PD-L1 immunotherapy (atezolizumab) and chemotherapy (mFOLFIRINOX), were found to induce substantial neoantigen-specific T-cell responses in patients with surgically resected PDAC, leading to prolonged recurrence-free survival. These results suggest that such combination therapy can effectively stimulate durable immune responses and may significantly delay disease recurrence in PDAC patients [36].

### 2.2. CTLA-4

Clinical trials investigating the role of CTLA-4 blockade in PDAC have produced promising yet complex results. CTLA-4, a potent immunoregulatory molecule, downregulates T-cell activation and inhibits antitumor immune responses, making it a target for cancer immunotherapy [37]. Bengsch et al. [38] demonstrated that the CTLA-4/CD80 pathway regulates T-cell infiltration in PDAC. Their study revealed that blocking CTLA-4 or CD80 stimulates CD4^+^ but not CD8^+^ T-cell infiltration, suggesting distinct mechanisms for the exclusion of CD4^+^ and CD8^+^ T-cells in PDAC. Additionally, high CTLA-4 expression has been associated with poor prognosis. Higher expression of CTLA-4 on CD8^+^ T-cells is significantly associated with a shorter overall survival in patients with metastatic PDAC, highlighting the potential impact of CTLA-4-mediated immunosuppression on disease prognosis [39,40].

Ipilimumab, an anti-CTLA-4 antibody, has shown potential to enhance T-cell responses and elicit antitumor immunity in various cancers, including melanoma and prostate cancer [41]. However, a phase 2 trial by Royal et al. [42] evaluated the efficacy of ipilimumab in treating locally advanced or metastatic PDAC and found no responders according to RECIST criteria. Nevertheless, one patient experienced a significant delayed response, suggesting that immunotherapeutic approaches to PDAC warrant further exploration despite limited immediate efficacy.

Combining ipilimumab with other treatments has shown potential benefits. A study demonstrated that combining ipilimumab with a GM-CSF-secreting cell-based vaccine (GVAX) in previously treated pancreatic cancer patients resulted in improved overall survival and mesothelin-specific T-cell responses [43]. Similarly, a phase 1b study by Kamath et al. (2020) evaluated ipilimumab and gemcitabine in advanced PDAC, establishing a safe regimen and suggesting that the combination may enhance the durability of response compared to gemcitabine alone [42]. Another study indicated that CTLA-4 blockade combined with the GVAX vaccine significantly enhances T-cell responses, diversifies T-cell receptor repertoires, and improves the overall survival in PDAC, suggesting that CTLA-4 inhibition can effectively potentiate antitumor immunity and overcome treatment resistance [43]. In summary, while CTLA-4 blockade alone shows limited efficacy in PDAC, these clinical trials suggest that combination therapies offer potential benefits for enhancing antitumor immune responses and improving patient outcomes.

## 3. AI in Detecting and Monitoring Immunotherapy Responses

**Figure 2:**
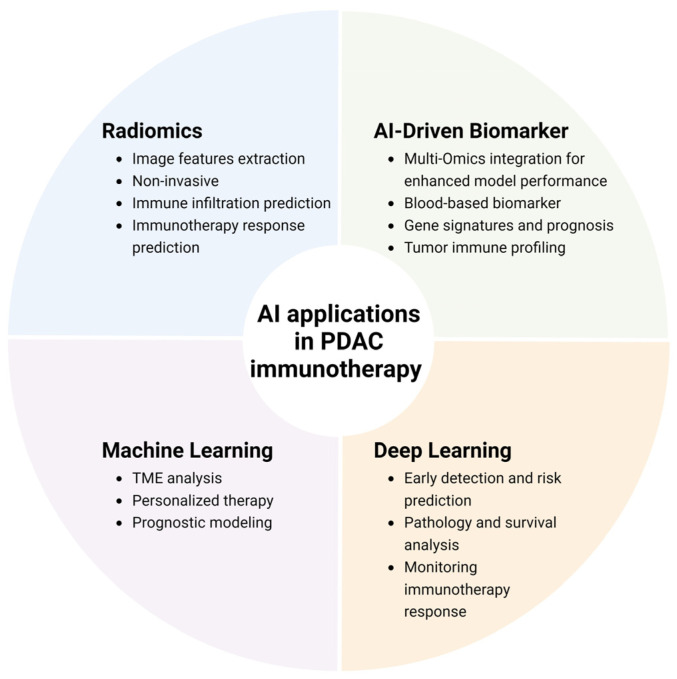
AI applications in PDAC immunotherapy. AI applications in PDAC immunotherapy include radiomics for non-invasive imaging analysis, biomarkers integrated with multi-omics for enhanced model accuracy, machine learning for personalized treatment strategies, and deep learning for risk prediction and monitoring.

PDAC remains one of the most challenging malignancies to detect and treat, largely due to its aggressive nature and the complexity of its tumor microenvironment (TME). Early detection is crucial but difficult to achieve through traditional methods, highlighting the need for innovative approaches. In this context, AI has emerged as a transformative tool, offering novel strategies to enhance the detection and monitoring of immunotherapy responses in PDAC (Figure 2).

### 3.1. The Need for AI in PDAC Detection and Monitoring

Conventional methods for monitoring PDAC, such as biopsies, are often invasive and pose risks to patients, while blood-based biomarkers like CA19-9 and carcinoembryonic antigen (CEA), although non-invasive, tend to be non-specific and may not fully capture the complex dynamics of the tumor microenvironment (TME), which is critical in determining immunotherapy outcomes [44,45,46]. On the other hand, imaging biomarkers, such as those derived from MRI, offer non-invasive alternatives but still require advanced techniques for accurate interpretation. AI, with its capacity to analyze vast and intricate datasets, can be applied to both blood-based and imaging biomarkers, offering a more precise approach to biomarker discovery and disease monitoring [47]. By integrating data from multiple sources, AI enhances our ability to understand and monitor PDAC, ultimately leading to more personalized and effective treatment strategies.

### 3.2. AI-Driven Improvement in Biomarkers

Blood-based biomarkers are essential for understanding PDAC progression and guiding treatment strategies. For example, the p53 biomarker, a tumor suppressor protein, is crucial in regulating cell division and preventing tumor formation. Mutations in the TP53 gene, which encodes the p53 protein, are frequently associated with various cancers, including PDAC [44,45,46]. Detecting these mutations is essential for understanding the disease’s progression and prognosis. AI-driven approaches; however, offer the potential to revolutionize this process. By leveraging advanced algorithms to analyze radiology and histopathology images, AI can identify patterns and biomarkers predictive of treatment response [48]. A recent study introduced a model-driven multi-modal deep learning approach that leverages a spiral transformation algorithm to effectively utilize 3D information and enhance data quality [49]. This innovative method significantly improved the non-invasive prediction of TP53 mutations, offering an alternative for PDAC detection and monitoring. Iwatate et al. demonstrated that radiogenomic analysis using CT imaging features can effectively predict p53 mutations and PD-L1 expression in PDAC, providing a non-invasive method to assess these critical biomarkers for prognostic evaluation [50].

In addition to blood-based biomarkers, broader AI applications in PDAC have led to the development of gene signatures beyond circulating biomarkers. For instance, a recent machine learning-based study created a prognostic gene signature (DPIRG) for PDAC, identifying immune biomarkers (e.g., PLEC, TRPV1) and potential drugs, such as thalidomide, that could convert cold tumors to more immunogenic states, thereby enhancing patient stratification and expanding immunotherapy options [51].

### 3.3. Radiomics-Based Prediction of Immunotherapy Response in PDAC

Radiomics, the high-throughput extraction of quantitative features from medical images [52], has emerged as a powerful tool in evaluating and treating PDAC, particularly in the context of immunotherapy [53]. The TME of PDAC is notoriously complex, often hindering the development of reliable predictive biomarkers for targeted therapies. Radiomics offers a non-invasive method to assess the TME and predict immune infiltration, providing crucial insights into potential responses to immunotherapy [54,55]. However, monitoring the efficacy of ICI treatment remains challenging due to phenomena like pseudoprogression, where tumors initially appear to grow before responding to treatment. Traditional criteria, such as RECIST and its modified versions like iRECIST, rely primarily on a change in the tumor size and often fail to account for these atypical response patterns. Even with improvements like imRECIST, the issue persists, highlighting the need for more advanced methods.

Recent studies have demonstrated the potential of radiomics to non-invasively assess the TME and predict immune cell infiltration. For instance, a machine learning classifier based on non-contrast MRI was developed to preoperatively predict CD8^+^ T-cell expression in PDAC patients, showing significant discriminative ability with an AUC of 0.89 in the training cohort and 0.69 in the validation cohort [56]. Pan et al. (2019) utilized MRI to monitor therapeutic responses in PDAC, demonstrating that specific radiomic features correlated with patient outcomes, thereby validating MRI’s utility in tracking immunotherapy efficacy [57]. Similarly, Eresen et al. (2020) conducted an early prediction study that showcased the use of MRI radiomics to predict responses to immunotherapy in PDAC, suggesting that early radiomic markers might serve as valuable tools in personalized treatment planning [8]. Additionally, Bian et al. (2022) constructed a machine learning model using preoperative radiomic features to evaluate tumor biology and predict postoperative outcomes, illustrating the predictive ability of radiomics in clinical settings [56]. Most recently, an investigation by Lu et al. (2024) developed a radiomics nomogram to predict the prognosis of PDAC patients undergoing immunotherapy, finding that their model could accurately stratify patients based on predicted outcomes [58]. Collectively, these studies underscore the critical role of radiomics in advancing the precision of PDAC immunotherapy.

### 3.4. Machine Learning Applications for PDAC Immunotherapy

Machine learning (ML) has emerged as a powerful tool in enhancing the efficacy of immunotherapy for PDAC. By leveraging complex datasets, ML models can uncover intricate biological mechanisms and predict treatment outcomes, thereby aiding in the development of personalized treatment strategies. Applications in this area include TME analysis, treatment response prediction, and prognostic assessments, among others.

For TME analysis, where ML’s ability to decode multifaceted immune interactions is crucial, ML models trained on over 1000 TME features from PDAC patients have been applied to predict treatment response and disease-free survival (DFS) following neoadjuvant anti-CD40 therapy. These models revealed that anti-CD40 therapy reduces T-cell exhaustion and is associated with increased CD44^+^CD4^+^ Th1 cells, which correlate with improved DFS outcomes [59]. Additionally, a metabolism-derived signature (MBS) developed via ML predicted immunotherapy outcomes by identifying connections with immune-resistant pathways and antitumor immunity. The analysis of data from 1188 patients underscored the significance of the metabolic landscape in shaping the TME, offering potential therapeutic targets for personalized PDAC treatment [60].

For treatment response prediction, ML’s capacity to discern patient-specific resistance mechanisms enables tailored therapies. ML plays a crucial role in differentiating resistance mechanisms in PDAC during PD-1 blockade therapy. Findings have indicated that increased MHC-I expression in malignant cells, combined with MHC and PD-1/PD-L suppression in CD8+ T-cells, is linked to nonresponse, underscoring ML’s potential to predict cellular-level treatment outcomes [61]. In another study, ML models based on routine hematologic and biochemical parameters demonstrated a high prediction efficiency for PD-1 combination therapy efficacy, with the AdaBoost classifier particularly effective in early therapeutic response prediction [62].

For prognosis, ML’s strength in handling large-scale gene expression data aids in robust survival prediction. A study utilizing LASSO, XGBoost, and Random Forest developed a risk signature linked to hypoxia and lactylation to predict PDAC prognosis and immunotherapy response, identifying CENPA as a promising therapeutic target [63]. Furthermore, a 12-gene prognostic signature based on naive B-cell-related genes was constructed using CIBERSORT and scRNA-seq data to investigate tumor-infiltrating immune cell interactions within the TME. This model, validated across multiple cohorts, demonstrated robust predictive power and highlighted notable immune infiltration differences between high- and low-risk groups, providing insights for immunotherapeutic strategies and individualized treatment plans for PDAC [64].

Machine learning is a versatile tool in PDAC immunotherapy, driving advancements in TME analysis, treatment response prediction, and prognostic modeling. Despite its promise, challenges remain, including the need for extensive, high-quality datasets and the effective integration of ML findings into clinical practice. Furthermore, the complexity of tumor biology and patient response variability calls for the continual refinement of ML models to enhance their accuracy and generalizability across diverse patient populations.

### 3.5. Deep Learning-Based Surveillance of Risk, Early Detection, and Immunotherapy Response/Outcomes of PDAC

Deep learning, a basis of AI, involves training artificial neural networks with multiple layers to learn complex patterns from large datasets. In cancer research, deep learning has become a powerful tool, enabling the analysis of vast amounts of biomedical data to uncover insights that were previously unattainable. Its ability to detect subtle patterns in medical images, genomic data, and other complex datasets has significantly advanced the field, leading to more accurate diagnostics, personalized treatment strategies, and a deeper understanding of cancer biology [65]. Unlike radiomics, which can be challenging to standardize, validate, and reproduce across different patients and imaging conditions [66], deep learning offers the advantage of better generalization and transferability, potentially improving the reliability and accuracy of biomedical analyses.

Early detection is particularly significant in PDAC due to its typically late diagnosis and poor prognosis. Early detection can significantly improve the efficacy of immunotherapy by identifying tumors at a stage where they are more likely to respond to treatment. One significant advancement is the use of deep learning models to analyze disease trajectories and predict pancreatic cancer risk. A study utilizing data from millions of patients in Denmark and the United States demonstrated that machine learning models could predict cancer occurrence with high accuracy, achieving an AUROC of 0.88 for predictions within 36 months [67]. This capability is instrumental in designing surveillance programs for high-risk patients, potentially improving early detection and patient outcomes. Additionally, a deep learning model, PANDA, was developed to non-invasively detect and classify PDAC using non-contrast Computed Tomography (CT). Trained on a dataset of 3208 patients, PANDA achieved an AUC of 0.986–0.996 in a multicenter validation, demonstrating high accuracy for PDAC detection. This model offers a promising tool for the large-scale, non-invasive screening and early detection of PDAC [68].

The application of AI in pathology and clinical analysis has significantly advanced the prediction of patient outcomes and the development of personalized treatment strategies in PDAC. AI-powered pathology slide analyzers, such as Lunit SCOPE IO, have been utilized to assess tumor-infiltrating lymphocytes (TILs) and classify immune phenotypes, demonstrating that higher intratumoral TIL densities correlate with a better prognosis in PDAC patients [69]. AI-based comprehensive analyses have integrated immune cell profiling with cancer stem cells (CSCs) and tumor budding (TB) to predict patient survival, outperforming traditional tumor–node–metastasis staging models [70]. The integration of radiology, pathology, and genomics data through AI has also been explored to predict PD-L1 expression and the tumor microenvironment, enhancing the selection of patients likely to benefit from immunotherapy [71].

Using deep learning based on image biomarkers to monitor the immunotherapy response has shown significant potential. A deep-learning algorithm, OrganoIDNet, was developed to analyze live-cell imaging of PDAC organoids, accurately detecting responses to chemotherapy and immunotherapy in real time. The study demonstrated the enhanced tumor-killing effects of PBMCs in organoid co-cultures with the PD-L1 inhibitor Atezolizumab, highlighting the platform’s potential for the dynamic assessment of the treatment efficacy in patient-derived PDAC models [72]. An ensemble deep learning model was developed using preoperative clinical and CT data to predict postoperative survival in PDAC patients, showing superior performance in predicting 1-year recurrence-free survival and comparable performance in predicting 2-year overall survival relative to the AJCC staging system, suggesting a similar potential in PDAC immunosurveillance [73]. Overall, AI’s application in PDAC immunotherapy surveillance encompasses a wide range of tools and methodologies, from digital pathology and deep learning models to integrated multi-omics analyses, all contributing to more precise and personalized treatment strategies.

## 4. Future Directions

The integration of AI in the detection and monitoring of PDAC represents a significant advancement in precision oncology. However, several challenges remain that must be addressed to fully realize the potential of AI in this field. One of the primary limitations is the efficacy of ICIs as a monotherapy for PDAC, which is characterized by a cold tumor microenvironment. This environment is typically resistant to immunotherapy, highlighting the need for innovative strategies to either transform PDAC into a more immunogenic, “hot” tumor or to develop effective combination therapies [19,28]. Future research should focus on enhancing the efficacy of single ICI treatments and exploring synergistic combinations that could overcome the immunosuppressive nature of PDAC.

Another significant challenge lies in the areas of data privacy and model explainability. As AI systems increasingly rely on patient data to make clinical predictions, ensuring the confidentiality and security of these data is paramount. Furthermore, the black-box nature of many AI models presents a barrier to their widespread adoption in clinical settings. Future studies should prioritize the development of transparent AI models that not only offer a high accuracy but also provide interpretable results that can be easily understood by clinicians. This will be crucial for gaining the trust of healthcare providers and ensuring that AI tools are effectively integrated into routine clinical practice.

To advance the application of AI in PDAC, future research should focus on integrating multi-omics and multi-modal data to enhance the precision of immunotherapy. Combining genomic, proteomic, and imaging data can provide a more comprehensive view of the tumor microenvironment and its response to treatment, leading to more personalized and effective therapeutic strategies. Additionally, longitudinal studies are needed to assess the long-term impact of AI-driven interventions on patient outcomes. These studies will be essential for understanding the durability of AI’s predictive capabilities and its role in guiding long-term treatment decisions.

Revolutionary technologies such as Large Language Models (LLMs) and Large Vision Models (LVMs) offer promising solutions to some of the current challenges in AI-driven oncology [74,75,76]. These models, pre-trained on vast amounts of data, can potentially be fine-tuned on domain-specific datasets to improve both the precision and efficiency of AI applications in PDAC. Tailoring these models to the specific needs of oncology could reduce the time and cost associated with training AI systems, making precision oncology more accessible and effective. Future research should explore the development and application of such tailored models, with a focus on optimizing their performance for clinical use.

## 5. Conclusions

AI has emerged as a transformative tool in the detection and monitoring of PDAC, offering novel approaches to overcome the limitations of traditional methods. The ability of AI to analyze complex datasets and uncover subtle patterns has significantly advanced our understanding of PDAC, leading to more accurate diagnostics and personalized treatment strategies. However, the application of AI in PDAC is still in its early stages, and several challenges must be addressed to fully harness its potential. These include improving the efficacy of ICIs, ensuring data privacy, enhancing model explainability, and integrating multi-omics data.

The future of AI in PDAC research lies in its ability to adapt and evolve with emerging technologies. Integrating multi-modal data, conducting longitudinal studies, and developing revolutionary AI models tailored to oncology will be crucial steps in advancing precision oncology. As these technologies continue to mature, they will undoubtedly play an increasingly central role in the fight against PDAC, offering hope for improved patient outcomes and the eventual transformation of this challenging disease into a more manageable condition. Continued collaboration between researchers, clinicians, and technologists will be essential to achieve these goals and realize the full potential of AI in cancer care.

## Figures and Tables

**Table 1:** Clinical trials involving immune checkpoint inhibitors (ICIs) for PDAC therapy (the data on clinical trials were obtained from ClinicalTrials.gov and accessed as of 15 April 2024).

ICIs Type	ICIs Name	Other Treatments	NCT	Phase	Status
CTLA-4	Ipilimumab	KRAS peptide vaccine	NCT04117087	PHASE1	RECRUITING
	Niraparib + Ipilimumab		NCT03404960	PHASE1|PHASE2	ACTIVE_NOT_RECRUITING
PD-1	Niraparib + Nivolumab		NCT03404960	PHASE1|PHASE2	ACTIVE_NOT_RECRUITING
	Nivolumab	BMS-813160, Gemcitabine, Nab-paclitaxel, Biopsy, Peripheral blood	NCT03496662	PHASE1|PHASE2	ACTIVE_NOT_RECRUITING
		Stereotactic Body Radiation (SBRT), CCR2/CCR5 dual antagonist, GVAX	NCT03767582	PHASE1|PHASE2	RECRUITING
		Irreversible Electroporation (IRE), Toll-Like Receptor 9	NCT04612530	PHASE1	RECRUITING
		KRAS peptide vaccine	NCT04117087	PHASE1	RECRUITING
		Albumin-bound paclitaxel, Paricalcitol, Cisplatin, Gemcitabine	NCT02754726	PHASE2	ACTIVE_NOT_RECRUITING
		BMS-986416	NCT04943900	PHASE1	ACTIVE_NOT_RECRUITING
		RO7496353, Capecitabine, S-1, Oxaliplatin, Nab-paclitaxel, Gemcitabine	NCT05867121	PHASE1	RECRUITING
		Daratumumab, KRAS vaccine	NCT06015724	PHASE2	RECRUITING
		Fluorouracil, Irinotecan, Irinotecan Hydrochloride, Leucovorin, Leucovorin Calcium, Oxaliplatin, Therapeutic Conventional Surgery	NCT03970252	EARLY_PHASE1	ACTIVE_NOT_RECRUITING
		Regorafenib, (Stivarga, BAY73-4506)	NCT04704154	PHASE2	ACTIVE_NOT_RECRUITING
		SX-682	NCT04477343	PHASE1	RECRUITING
	Pembrolizumab	Defactinib	NCT03727880	PHASE2	RECRUITING
		PEGPH20	NCT03634332	PHASE2	UNKNOWN
		GEN1042, Cisplatin, Carboplatin, 5-FU, Gemcitabine, Nab paclitaxel, Pemetrexed, Paclitaxel	NCT04083599	PHASE1|PHASE2	RECRUITING
		Folfirinox	NCT05132504	PHASE2	RECRUITING
		BXCL701	NCT05558982	PHASE2	RECRUITING
		Olaparib	NCT04666740	PHASE2	RECRUITING
		Lenvatinib Mesylate	NCT04887805	PHASE2	RECRUITING
		Belzutifan, Lenvatinib	NCT04976634	PHASE2	RECRUITING
		Imiquimod, Sotigalimab, Synthetic Tumor-Associated Peptide Vaccine Therapy, Computed Tomography, Magnetic Resonance Imaging	NCT02600949	PHASE1	RECRUITING
		Epacadostat	NCT03432676	PHASE2	WITHDRAWN
		Lenvatinib	NCT05273554	PHASE1	RECRUITING
		PF-07934040, Gemcitabine, Nab-paclitaxel, Cetuximab, Fluorouracil, Oxaliplatin, Leucovorin, Bevacizumab, pemetrexed, Cisplatin, Paclitaxel, Carboplatin	NCT06447662	PHASE1	NOT_YET_RECRUITING
		Nab-paclitaxel, Gemcitabine, Cisplatin, Irinotecan, Capecitabine, Olaparib	NCT04753879	PHASE2	RECRUITING
		Epacadostat, Oxaliplatin, Leucovorin, 5-Fluorouracil, Gemcitabine, nab-Paclitaxel, Carboplatin, Paclitaxel, Pemetrexed, Cyclophosphamide, Carboplatin, Cisplatin, 5-Fluorouracil, investigator’s choice of platinum agent	NCT03085914	PHASE1|PHASE2	COMPLETED
		Futibatinib, Cisplatin, 5-FU, Oxaliplatin, Leucovorin, Levoleucovorin, Irinotecan	NCT05945823	PHASE2	RECRUITING
PD-L1	Atezolizumab	PEGPH20	NCT03979066	PHASE2	TERMINATED
		Tumor Treating Fields, Gemcitabine, Nab-paclitaxel	NCT06390059	PHASE2	RECRUITING
		RO7496353, Capecitabine, S-1, Oxaliplatin, Nab-paclitaxel, Gemcitabine	NCT05867121	PHASE1	RECRUITING
		Autogene cevumeran, mFOLFIRINOX	NCT05968326	PHASE2	RECRUITING
		Nab-paclitaxel, Gemcitabine, Oxaliplatin, Leucovorin, Fluorouracil, Cobimetinib, PEGPH20, BL-8040, Selicrelumab, Bevacizumab, RO6874281, AB928, Tiragolumab, Tocilizumab	NCT03193190	PHASE1|PHASE2	ACTIVE_NOT_RECRUITING
	Durvalumab	Rintatolimod	NCT05927142	PHASE1|PHASE2	RECRUITING
